# Massive Retinal Gliosis in Microphthalmic Globe With Cyst Masquerading as a Neoplasm

**DOI:** 10.7759/cureus.15419

**Published:** 2021-06-03

**Authors:** Ruchi Goel, Shweta Raghav, Ayushi Agarwal, Ravindra Saran, Akash Raut

**Affiliations:** 1 Ophthalmology, Maulana Azad Medical College, New Delhi, IND; 2 Pathology, Govind Ballabh Pant Institute of Postgraduate Medical Education and Research, Maulana Azad Medical College, New Delhi, IND

**Keywords:** microphthalmia, massive retinal gliosis, microphthalmia with cyst, apparent diffusion coefficient, glial fibrillary acid protein

## Abstract

We report a rare case of a bilaterally blind, 33-year-old male, who presented with rapidly progressive, painless swelling in the right orbit for two months, in the absence of ocular trauma. Suspecting an intraocular neoplasm, a contrast-enhanced MRI (CE-MRI) orbit was performed, which was suggestive of a right-sided superomedial eccentric soft-tissue lesion with bilateral rudimentary globes associated with cyst, hypoplastic optic nerves, and focal areas of calcification. Diffusion-weighted MRI demonstrated diffusion restriction and yielded an indeterminate value of apparent diffusion coefficient (ADC). A right enucleation with excision of the cyst was performed. Histopathological examination confirmed the diagnosis of a right-sided massive retinal gliosis (MRG) with bilateral microphthalmia with cyst. This case demonstrates the role of a detailed histopathological analysis along with immunohistochemistry (IHC) in differentiating MRG from a neoplasm.

## Introduction

Microphthalmia with cyst is a relatively common congenital entity, which results from developmental arrest during the sixth to seventh week of gestation at the 7-14-mm stage of embryological growth [1]. Massive retinal gliosis (MRG), originally described by Von Hippel in 1905, represents a pseudoneoplastic, polyclonal proliferation of retinal glial cells [2]. It is more common in children as compared to adults and is rarely seen in association with microphthalmia with cyst [3]. In this report, we report a case of an adult male with bilateral microphthalmia with cyst presenting with a sudden, rapidly growing mass on the right side, which was later diagnosed as MRG on the basis of histopathological and immunohistochemistry (IHC) studies.

## Case presentation

**Figure 1:**
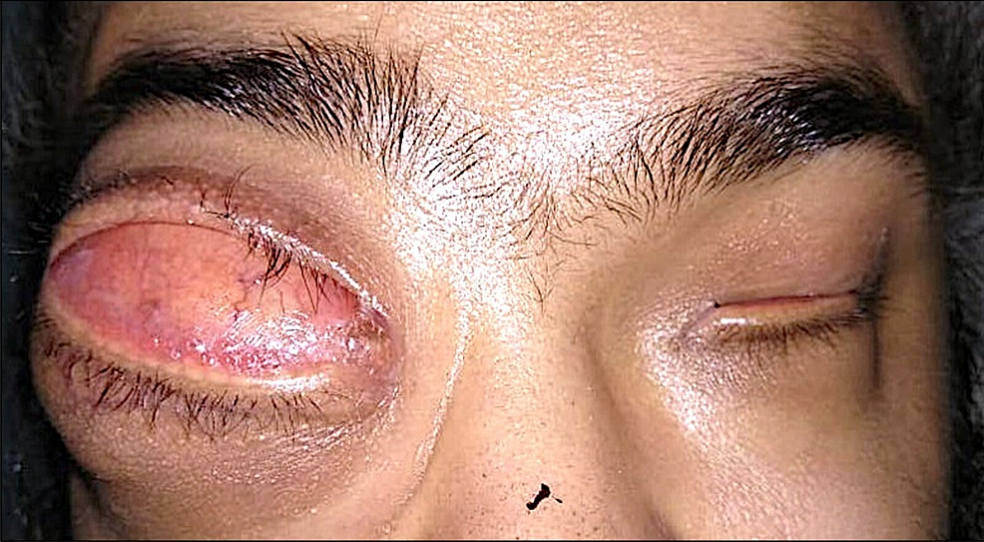
Clinical photograph showing a right-sided mass

A 33-year-old male, bilaterally blind since birth, presented with a sudden, painless right orbital mass and inability to open the right eye for the past two months (Figure 1).

There was no history of trauma, radiation, or infection. On palpation, the mass was firm and transillumination-negative. Clinically, the left socket appeared anophthalmic with a pea-sized mass, positive on transillumination. Systemic examination was unremarkable. In light of the rapid growth and absence of trauma, a provisional diagnosis of right intraocular malignant neoplasm was made.

**Figure 2:**
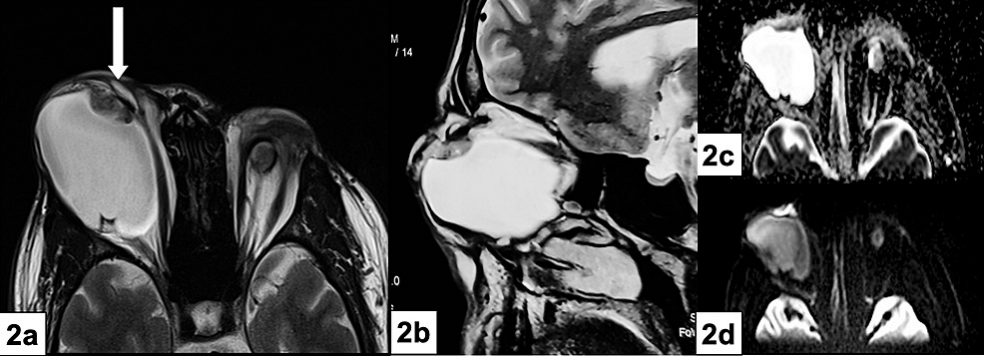
Contrast-enhanced MRI orbit a) T1-weighted MRI showing bilateral microphthalmic globe with cystic lesions on both sides and an eccentric soft-tissue lesion on the right side (white arrow). b) T2-weighted MRI showing the isointense soft tissue lesion on the superomedial aspect of the right cyst. c) Diffusion-weighted MRI demonstrating hyperintense area with d) corresponding ADC map showing diffusion restriction MRI: magnetic resonance imaging; ADC: apparent diffusion coefficient

A contrast-enhanced MRI (CE-MRI) orbit with a T1-weighted fat-suppressed sequence showed bilateral hypoplastic globes with hyperintense cystic lesions. The cysts measured 6.1 x 4.2 x 4.1 cm and 1.6 x 1.5 x 1.6 cm on the right and left sides, respectively. The T2-weighted image revealed an eccentric soft tissue component, measuring 1.6 x 0.8 cm, on the superomedial aspect of the right cyst, which was isointense to extraocular muscles. Diffusion-weighted imaging (DWI) demonstrated a hyperintense area with an apparent diffusion coefficient (ADC) value of 1.23 x 10^-3^ mm^2^/s. The hypointense region on the corresponding ADC map demonstrated diffusion restriction (Figure 2).

**Figure 3:**
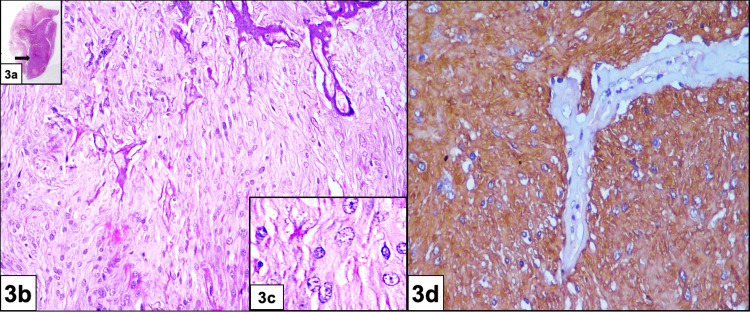
Histopathological examination and immunohistochemistry Histopathological examination of a) solid area (2X black arrow scanner view) revealing b) spindle-shaped cells with eosinophilic fibrillary cytoplasm and prominent dilated blood vessels (20X), c) better delineated at higher power (40X). d) Immunohistochemistry demonstrating strong positivity for glial fibrillary acidic protein (GFAP)

A right enucleation with excision of the mass was performed. The cystic cavity was found to be filled with blood and the wall had solid areas. Histopathological examination of the solid area revealed spindle-shaped glial cells arranged in interlacing short fascicles with abundant eosinophilic fibrillary cytoplasm and numerous dilated blood vessels. IHC showed intense positivity for glial fibrillary acidic protein (GFAP), confirming astroglial origin (Figure 3).

**Figure 4:**
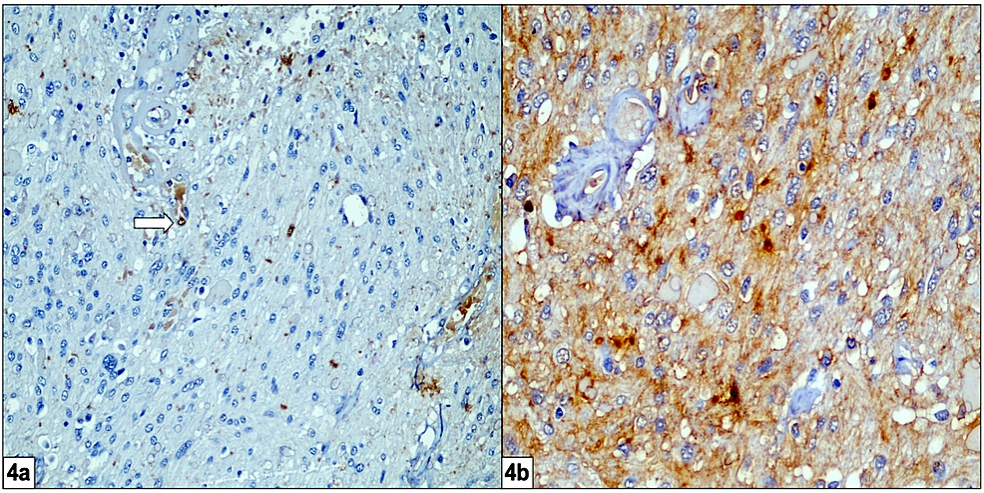
a) MIB-1 labeling index of less than 2% (white arrow). b) Immunohistochemistry showing epithelial membrane antigen (EMA) positivity

Positive IHC staining for epithelial membrane antigen (EMA) and a MIB-1 index of less than 2% were also noted (Figure 4).

A diagnosis of bilateral microphthalmia with cyst with right MRG was made. Postoperatively, the patient underwent an uneventful recovery and was referred for cosmetic rehabilitation. The patient was bilaterally blind and ultimately opted for a spectacle prosthesis.

## Discussion

MRG represents a benign, intraocular, reactive proliferation of glial tissue occurring in response to variable insults. The reported inciting factors include chronic inflammation, trauma, vascular abnormalities, glaucoma, and retinal diseases such as long-standing retinal detachment and retinopathy of prematurity [4,5]. In our case, the presentation was late in onset and no triggering events were found.

ADC, derived from DWI-MRI, is used as an adjunct to differentiate malignant lesions from benign ones. In reactive gliosis, altered cell membranes, damage to myelin, and axonal loss contribute to the restriction of water motion and result in high ADC values (ranging from 1.65 to 1.88 x 10^-3^ mm^2^/s) [6]. In our case, however, DWI yielded indeterminate ADC values, and hence a malignant lesion could not be ruled out.

The benign nature of the lesion was eventually established on the basis of intense glial positivity and a MIB-1 index of less than 2% on histopathology. Histopathology along with IHC markers also helped in ruling out other possible neoplastic entities such as astrocytoma, schwannoma, teratoma, ependymoma, metastasis, retinal hemangioblastoma, vasoproliferative tumors of the retina, and melanoma [2,7]. 

Both MRG and low-grade pilocytic astrocytoma (glioma) may possess a relatively higher rate of MIB-1 index, thereby posing a diagnostic challenge. Differentiating features of low-grade astrocytoma include the presence of elongated eosinophilic processes, typically known as Rosenthal fibers (which are less commonly seen in MRG), p53 and BRAF gene mutations, and early involvement of the optic nerve, usually within the first decade of life. Almost one-third of the glioma cases seen may be associated with neurofibromatosis type 1 (NF1) [2,8]. Vasoproliferative tumors (VPT), unlike glial-predominant MRG, demonstrate proliferation of glial as well as vascular components on histopathology. Ependymoma is a glial tumor of the central nervous system. Histologically, the presence of pseudorosettes, ependymal differentiation, and luminal dot-like positivity for EMA on IHC along with a low MIB-1 index facilitates the diagnosis of an ependymoma [9]. In rare cases, an orbital teratoma presents as sudden-onset proptosis and appears variegated on palpation. Usually found in association with a developmentally sound eye, microphthalmia is not uncommon. Histologically, the presence of three germ layers, surface ectoderm, neuroectoderm, mesoderm, or endoderm, is characteristic of a teratoma [10]. On imaging, it appears as a multi-loculated cystic mass with associated fat and ossification [11]. Although MRG exhibits certain overlapping histological features with schwannoma, findings such as Antoni A and B cells with palisading of the nuclei, Verocay bodies, and the presence of intercellular reticulin favor the diagnosis of the latter [2].

We hypothesize that in our case, the rupture of intralesional blood vessels led to the sudden increase in the size of the cyst, and the MRG contributed to the firm consistency.

## Conclusions

MRG associated with microphthalmia with cyst is an uncommon presentation and should be considered in the differential diagnoses of a sudden, rapid increase in the size of microphthalmia with cyst. This case also highlights the importance of utilizing IHC markers in establishing the benign nature of the mass that obviated the need for unnecessary imaging for metastatic workup.
